# High-throughput expression of animal venom toxins in *Escherichia coli* to generate a large library of oxidized disulphide-reticulated peptides for drug discovery

**DOI:** 10.1186/s12934-016-0617-1

**Published:** 2017-01-17

**Authors:** Jeremy Turchetto, Ana Filipa Sequeira, Laurie Ramond, Fanny Peysson, Joana L. A. Brás, Natalie J. Saez, Yoan Duhoo, Marilyne Blémont, Catarina I. P. D. Guerreiro, Loic Quinton, Edwin De Pauw, Nicolas Gilles, Hervé Darbon, Carlos M. G. A. Fontes, Renaud Vincentelli

**Affiliations:** 1 Unité Mixte de Recherche (UMR) 7257, Centre National de la Recherche Scientifique (CNRS) Aix-Marseille Université, Architecture et Fonction des Macromolécules Biologiques (AFMB), Marseille, France; 2 CIISA-Faculdade de Medicina Veterinária, Universidade de Lisboa, Avenida da Universidade Técnica, 1300-477 Lisbon, Portugal; 3 NZYtech Genes & Enzymes, Campus do Lumiar, Estrada do paço do Lumiar, 1649-038 Lisbon, Portugal; 4 Institute for Molecular Bioscience, The University of Queensland, St Lucia, 4072 Australia; 5 Mass Spectrometry Laboratory, B6c University of Liège, MolSys-Quartier Agora, Allée du six Aout 11, 4000 Liège, Belgium; 6 CEA/DRF/iBiTecS, Service d’Ingénierie Moléculaire des Protéines, 91191 Gif-sur-Yvette, France

**Keywords:** Venom peptides, Disulphide bonds, Periplasm, *Escherichia coli* (*E. coli*), Drug discovery library, High-throughput production

## Abstract

**Background:**

Animal venoms are complex molecular cocktails containing a wide range of biologically active disulphide-reticulated peptides that target, with high selectivity and efficacy, a variety of membrane receptors. Disulphide-reticulated peptides have evolved to display improved specificity, low immunogenicity and to show much higher resistance to degradation than linear peptides. These properties make venom peptides attractive candidates for drug development. However, recombinant expression of reticulated peptides containing disulphide bonds is challenging, especially when associated with the production of large libraries of bioactive molecules for drug screening. To date, as an alternative to artificial synthetic chemical libraries, no comprehensive recombinant libraries of natural venom peptides are accessible for high-throughput screening to identify novel therapeutics.

**Results:**

In the accompanying paper an efficient system for the expression and purification of oxidized disulphide-reticulated venom peptides in *Escherichia coli* is described. Here we report the development of a high-throughput automated platform, that could be adapted to the production of other families, to generate the largest ever library of recombinant venom peptides. The peptides were produced in the periplasm of *E. coli* using redox-active DsbC as a fusion tag, thus allowing the efficient formation of correctly folded disulphide bridges. TEV protease was used to remove fusion tags and recover the animal venom peptides in the native state. Globally, within nine months, out of a total of 4992 synthetic genes encoding a representative diversity of venom peptides, a library containing 2736 recombinant disulphide-reticulated peptides was generated. The data revealed that the animal venom peptides produced in the bacterial host were natively folded and, thus, are putatively biologically active.

**Conclusions:**

Overall this study reveals that high-throughput expression of animal venom peptides in *E. coli* can generate large libraries of recombinant disulphide-reticulated peptides of remarkable interest for drug discovery programs.

**Electronic supplementary material:**

The online version of this article (doi:10.1186/s12934-016-0617-1) contains supplementary material, which is available to authorized users.

## Background

Animal venoms contain a complex arsenal of disulphide-reticulated peptides that present an enormous structural and pharmacological diversity. The global animal venom library can be seen as a collection of more than 40,000,000 peptides of which only a very small fraction is known [1]. These molecules have been fine-tuned during the course of evolution to present not only target selectivity but also low immunogenicity and high stability [2]. The molecular targets of venom peptides are mainly present at the cell surface and are involved in various human health disorders such as pain, cancer, neurodegenerative diseases, cardio-vascular diseases, diabetes, obesity and depression [3, 4]. However, although the use of venoms for drug discovery is rapidly emerging, it is still mostly an unrealized prospect due to recurrent technical bottlenecks that represent venom exploration. The advent of -omic techniques [5–7] lead to an explosion of the number of toxin sequences known, which are available for biomedical and biotechnological exploitation [8]. High-throughput production of these highly biologically-relevant molecules using recombinant technologies is currently the major limiting step between sequencing and biological screening and represents nowadays a considerable challenge. To date, no comprehensive recombinant libraries of venom peptides are accessible for high-throughput screens (HTS) to identify novel therapeutics, as an alternative to synthetic chemical libraries.

Venom peptides generally contain between one to eight disulphide bonds which must be oxidized with the correct disulphide-bonding pattern in order to be active [9]. Production of recombinant peptides in *Escherichia coli* has a number of advantages over other biological systems, including a reduced cost, rapid growth, high biomass production, easily scalable cultivation and well-established regulations for therapeutic protein production. However, a priori, *E. coli* is not the ideal host to catalyse the formation of native disulphide bonds as its cytoplasm displays a particularly reducing environment [10]. Thus, proteins with disulphide bonds are especially prone to aggregation in *E. coli* as a result of mispaired intra- or inter-molecular disulphide bonds [11]. In addition, production of recombinant proteins in bacteria is regulated by strong promoters, which favour the accumulation of misfolded recombinant proteins in the form of insoluble aggregates or inclusion bodies. Failure in reaching the bioactive conformation increases with the number of cysteine residues, due to the number of possible isoforms but also to the complexity of disulphide bond patterns. To overcome these disadvantages we developed a system for the efficient production of disulphide-bond-containing proteins and peptides using a cleavable DsbC fusion in the strain BL21 (DE3) pLysS [12]. These studies were extended in the accompanying paper [13] that revealed, even if the peptide folding by DsbC occurs mainly ex vivo, that the expression of venom peptides when fused to DsbC in the bacterial periplasm lead to the production of milligram quantities of active toxins. The data also confirmed that release of fusion tags from recombinant peptides is effectively performed, even under non-optimal conditions, by TEV protease. These studies overall describe the development of an efficient high-throughput system for the expression and purification of disulphide-reticulated venom peptides in *E. coli*.

The main goal of the VENOMICS project was to replicate in vitro the diversity of animal venoms in order to generate large peptide banks that could be applied in pharmacological screens used in drug discovery programs. Starting from a bank of ~25,000 sequences, here a subset of ~5000 venom peptides with size of 35 or more residues, selected from the transcriptomics and proteomics analysis of natural venoms, representing the widest diversity in term of species, size, disulphide content and patterns, was selected for the production of a recombinant animal venom peptide library. To complete the VENOMICS peptide library, around 900 peptides (with size below 35 residues) were efficiently produced by chemical synthesis and oxidized (CEA, manuscript in preparation). The results of the nine-month production phase for the recombinant expression indicated that out of the 4992 venom peptides selected for recombinant expression, 2736 (55%) peptides could be produced using a single protocol in a soluble and oxidized form in quantities compatible with the drug discovery program. Recently, this unique bank of recombinant venom peptides was screened against therapeutic targets linked with diseases such as diabetes, obesity, inflammation and allergies, identifying dozens of novel drug leads. These data confirm that this unique library of animal venom peptides contains recombinant peptides that are both correctly folded and biologically active.

## Methods

### Gene synthesis and cloning

Genes encoding 4992 animal venom peptides originating from 201 different species, including terrestrial, marine and flying species, were designed for expression in *E. coli*. Additional file 1: Table S1 describes the animal groups from which the peptides were selected. The number of genes to synthesise (4992) was selected considering the production of 52 plates in 96-well format (52 × 96 = 4992). Venom genes were designed by back-translating the peptide sequence and optimizing codon usage for high levels of expression in *E. coli*, using the ATGenium codon optimization algorithm (NZYTech genes & enzymes, Portugal). Global codon usage considers codons used preferentially in highly expressed or average native *E. coli* genes and exclusion of naturally rare codons, as well as ensuring an equal proportion of the two cysteine codons. GC content was set to vary between 40 and 60%. Presence of G/C islands, which might promote frame-shifting, was minimized by selectively avoiding runs of consecutive G and/or C greater than 6 nucleotides. In addition, no contiguous string of nucleotides longer than five nucleotides was allowed. Genes were designed to ensure the absence of *E. coli* regulatory sequences such as promoters, activators or operators. Codon adaptation index (CAI) estimates were calculated as the geometric mean for test gene codons of the ratio of the codon frequency in highly expressed *E. coli* genes divided by that of the highest frequency codon for each amino acid in those genes. Genes were designed to ensure a CAI value higher than 0.8. Genes were engineered to encode the TEV protease cleavage site (ENLYFQ/) at the N-terminus of the target peptide sequence. This cleavage site was encoded by the sequence GAGAACCTGTACTTCCAA for all genes. The stop codon selected for all genes was TAA and was duplicated. The codon usage table used to design the DNA sequences of the 4992 genes is available in Additional file 2: Table S2.

Synthetic genes were produced in a high-throughput pipeline using previously optimized procedures. Briefly, genes were assembled from 40 to 60 bp oligonucleotides through PCR using KOD Hot Start DNA Polymerase (EMD Millipore). Oligonucleotides were designed using NZYOligo designer (NZYTech genes & enzymes, Portugal) to have a maximum length of 60 bp and to ensure a 20 bp gap between primers located in the same strand. Oligonucleotides were synthesised by Integrated DNA Technologies at the smallest scale with desalting purification. PCR reactions were performed in a volume of 50 µL in 96-well PCR plates. After amplification, assembled PCR products were purified using NZYGelpure 96 well plate kit (NZYTech genes & enzymes, Portugal) in a Tecan workstation (Switzerland). Purified PCR products (~50 ng) were subsequently cloned into pHTP4 expression vector using the NZYEasy Cloning kit (NZYTech genes & enzymes, Portugal), according to established protocols for ligation independent cloning (LIC) technology. In pHTP4 venom peptide genes are under the control of a T7 promoter. Recombinant venom peptide fusion proteins contain an N-terminal DsbC fusion (with a signal sequence to export the protein to the *E. coli* periplasmic space), an internal hexa-histidine tag for purification and a TEV recognition sequence to allow cleavage and isolation of the native peptide. Following the cloning reaction, recombinant plasmids were transformed using a high-throughput method into NZY5α competent *E. coli*. The transformed bacteria were spread on LB kanamycin plates. After overnight incubation at 37 °C, one colony per transformation was picked and grown in liquid media supplemented with 50 µg/mL of kanamycin in 24-deep-well-plates (5 mL) sealed with gas-permeable adhesive seals. The plasmids were purified from the bacterial pellets using NZYMiniprep 96 well plate kit (NZYTech genes & enzymes, Portugal) on a Tecan robot (Switzerland) and subsequently sequenced. In case the DNA sequence was not 100% identical to the designed gene, a second and eventually a third colony were picked for screening. All 4992 recombinant plasmids were completely sequenced in both directions to ensure consistency with the defined sequence.

### High-throughput venom peptide expression

All steps were carried out in 24-deep-well plates (DW24) with few modifications (see below) of the lab standard protocol [14, 15], which is described briefly below. 96 recombinant pHTP4 plasmids were used to transform BL21 (DE3) pLysS *E. coli* cells in 96-well format, at a time. For the production, in each DW24; 12 × 2 mL of auto-induction medium supplemented with kanamycin (50 μg/mL) and chloramphenicol (34 μg/mL) was inoculated (1/40 v/v) for each peptide. Downscaling the culture volume from 4 to 2 mL, with a better aeration, doubled the production yield compared to the previous protocol. The aim of the VENOMICS project is to produce a comprehensive library of peptides in HTS format (250 µL at 10 μM). Taking into account the final average peptide yield obtained with the periplasmic DsbC fusion, described in the accompanying paper [13], a scale of 12 × 2 mL culture seemed to be the best compromise between yields, labour time and cost. To express 96 peptides in parallel, a series of 48× DW24 were inoculated simultaneously (1152 cultures) and grown over 24 h at 25 °C in a Microtron shaking incubator (INFORS-HT, Switzerland) (600 rpm). The expression at 25 °C was preferred as it gave slightly higher peptide recovery yield than the previous procedure (37 °C for 4 h followed by 18 h at 17 °C). To be able to put 2 × 24 DW24 in the incubator simultaneously, a plexiglass second level was custom-made and added inside the incubator. The ZYP-5052 medium was replaced by the NZY Auto-Induction LB medium (NZYTech, genes & enzymes, Portugal) to gain in productivity, without any significant difference in protein yield (data not shown). At the end of the culture, the OD_600nm_ was 12, on average. Cells were collected by centrifugation and each well re-suspended in 0.5 mL of lysis buffer (50 mM Tris, 300 mM NaCl, 10 mM Imidazole, pH 8.0, 0.25 mg/mL Lysozyme) by 15 min shaking at 20 °C in a Microtron shaking incubator (INFORS-HT, Switzerland) (800 rpm). The DW24 plates were frozen at −20 °C.

### High-throughput protein purification by nickel affinity chromatography

12× DW24 (corresponding to the over-expression of 24 venom peptides) were thawed, in one go, for 10 min at 37 °C in a water-bath followed by 15 min shaking at 20 °C in a Microtron shaking incubator (INFORS-HT, Switzerland) (800 rpm). During this time the cultures were lysed by lysozyme and the solution became viscous. After the addition of 10 µg/mL DNAse and 20 mM MgSO_4_ in each well and another 10 min shaking at 20 °C, the 12 × 0.5 mL lysed bacteria for each peptide were pooled and transferred to a new DW24 to have 24 distinct 6 mL lysates of DsbC-His-peptide fusions per DW24. To ensure complete cell lysis, the DW24 was sonicated in a plate sonicator (Ultrasonic processor XL, Misonix Inc., USA) for 5 min (power 5, 30 s ON/OFF cycles). Purification was performed by immobilized metal affinity chromatography (IMAC) as described in the accompanying paper [13] where all the steps were automated on a Tecan Freedom EVO 200 robot (Switzerland) containing a vacuum manifold. Briefly, the 6 mL of crude cell lysates were incubated with 4 × 200 µL Ni^2+^ Sepharose 6 Fast flow resin (GE Healthcare, 17-5318-02) with bound Nickel and then transferred (4 wells per peptide) into 96-well filter plates (20 µm) (Macherey–Nagel). The wells were washed twice with 800 µL buffer A (50 mM Tris, 300 mM NaCl, 10 mM Imidazole, pH 8) followed by three washes with 800 µL of 50 mM Tris, 300 mM NaCl, 50 mM Imidazole, pH 8 buffer. The recombinant fusion proteins were eluted from the resin beads with 1 mL of elution buffer (50 mM Tris, 300 mM NaCl, 250 mM Imidazole, pH 8). Rather than collecting the elution in a 96 deep-well plate (DW96) like in the standard procedure, this DW plate was replaced by a DW24, pooling the 4 × 1 mL of elution of a single peptide into a single well of the DW24. This procedure was reproduced four times in one day in order to purify the 96 fusion proteins. The total time taken for a single round of this process was around 4 h.

### High-throughput TEV cleavage

On the same day, after the 96 purifications, the concentration of the 96 purified DsbC-His-fusion proteins was calculated spectrophotometrically (OD at 280 nm) in a micro-titre plate reader (Genios plus, TECAN, Switzerland). TEV_SH_ protease [16] (stored at 2 mg/mL in 20 mM Hepes, 300 mM NaCl, 10% Glycerol, pH 7.4 buffer, see accompanying paper [13] for the production protocol) was added (1/10 w/w) directly in the elution buffer by the Tecan robot. The final concentration of DTT was adjusted to 0.1 mM with fresh DTT while adjusting the final cleavage reaction volume to 5 mL with elution buffer. The 96 samples were then incubated overnight at 30 °C in a Microtron shaking incubator (INFORS-HT, Switzerland) (200 rpm) to allow total cleavage of the DsbC-His fusion protein.

### High-throughput target peptide purification by reverse phase chromatography

After overnight TEV cleavage, samples were acidified for 1 h with 5% acetonitrile, 0.1% formic acid (FA) by adding 500 µL of a 55% acetonitrile and 1.1% formic acid stock solution, at room temperature under mild agitation. At this step, TEV protease and mis-folded peptides were precipitated by the acidification and the 96 samples were subjected to the final purification step on C18 chromatographic resin. The C18 purification was performed using an automated Solid Phase Extraction (SPE) procedure specifically developed for this project and was implemented on a Tecan robot. First, the 4x DW24 containing the 96 acidified samples were centrifuged at room temperature (4000×*g* for 10 min) and the supernatants (5 mL) were transferred (5 × 1 mL with vacuum between each cycle to remove the unbound materials) into a 96-well filter plate (20 µm) (Macherey–Nagel) filled with 50 µL of C18 reversed phase beads (100 Å, Sigma 60756-50G) that had been activated in pure acetonitrile and equilibrated in solvent A (5% acetonitrile, 0.1% FA). After loading the samples, the C18 resin was washed twice with 800 µL of solvent A followed by one wash of 800 µL of solvent A without FA. The cleaved DsbC fusion partner is eliminated in the flow through and wash fractions of the SPE purification. The pure target peptides were eluted from the resin beads in 560 µL of elution buffer (50% acetonitrile/water). At the end of the SPE, the DW96 containing the target peptides in 560 µL of elution buffer (50% acetonitrile/water) were evaporated under a chemical hood with mild agitation over 16 h. Thus, the peptides were obtained in 280 µL of pure water (the more volatile acetonitrile having been evaporated). The peptides were stable for several weeks at 4 °C in these conditions (data not shown).

### High-throughput quality control and quantification by mass spectrometry

An aliquot (20 µL) was analysed in-house on a reverse phase C18 column at 37 °C (Hypersil GOLD 50 × 1.0 mm, 1.9 μm, 175 Å, ThermoScientific) at a flow rate of 200 μL/min on an ultra-high performance liquid chromatography-mass spectrometry (UHPLC-MS) with electrospray detection (Accela High Speed LC system with detector MSQ+ , ThermoScientific, San Jose, CA). The gradient slope (solvent A: water, B: acetonitrile, both solvents containing 0.1% formic acid) went from 5 to 40% B in 2 min followed by an 80% wash and re-equilibration (total time: 6 min). MS acquisition was performed with *m/z* ranging between 100 and 2000 Da. To confirm correct target peptide molecular weight, the resulting mass spectra were de-convoluted using manual calculations. The isotopic pattern measured was compared with the theoretical one determined from the amino acid sequences using Data Explorer software (version 4.9, Applied Biosystems). The quantitative calculation of target peptide yields were determined using automatic processing with Xcalibur software (ThermoScientific), by OD_280nm_ measurement and peak area integration.

Before freezing the bank in the micro-titer plate format, an aliquot of 10 µL was taken from the 260 µL of purified target peptides and frozen to have an external confirmation that the peptide oxidation states were correct and that the toxins were in the expected position in the plates. This analysis was done by ESI–MS (Micro-TOF, Bruker) and/or MALDI-MS (UltrafleXtreme, Bruker) at the University of Liege, Belgium.

### Venom peptide bank preparation for high-throughput screening

Since the final peptide quantification method was based only on the OD_280nm_ measurement and integration of peak areas on the UHPLC, and because we estimated that this quantification mode (the only one compatible with the throughput of the process) could lead to errors of up to 100% on the concentration for peptides with low molar extinction coefficients, we decided to divide the peptides into three subsets, based on concentration, for the screening process. When the peptide concentration was above 20 µM, the quantification was considered accurate and the peptide concentration was adjusted to 10 µM before aliquoting. When the concentration ranged from 5 to 20 µM, the peptides were aliquoted at this concentration. When the peptide concentration ranged from 1 to 5 µM, the peptides were aliquoted as such in a separate series of plates (see below). When the concentration was below 1 µM the peptides were discarded and counted as a non-producing clone. From this information, the Tecan robot, diluting with pure water, adjusted the most concentrated peptides (concentration >20 µM) to 250 µL at a final concentration of 10 µM in a DW96 plate. Each DW96 plate contained 80 target peptides and 2x8 empty wells for assay controls. From this original DW96 stock, the Tecan robot made 5 copies of microtiter plates containing 50 µL of target peptide. These plates are named the “10 µM bank”. The plates were shock frozen and stored at −80 °C until delivery to the screening laboratory (the delivery was done in 2 batches). Remaining concentrated peptide was shock frozen directly in the DW96 and kept as a backup for validation of potential hits, post-screening. When the peptide concentration ranged between 5 and 20 µM, the 250 µL left after the evaporation of the SPE fractions were reorganized in DW96 and treated as above to generate 5 × 50 µL plates. These plates were also called the “10 µM bank”. Finally, when the concentration was ranging from 1 to 5 µM, the peptides were reorganized in DW96 and treated as above to generate 5 × 50 µL plates. These plates were called the “1 µM bank”. The acquisition of the LC–MS data, analysis of the spectra, the Tecan concentration adjustment and aliquoting phases took less than 24 h for 96 peptides. The final validation of the bank by ESI–MS and/or MALDI-MS was confirmed by University of Liège.

## Results

The time frame allocated within the VENOMICS project to establish a large recombinant, active, disulphide-reticulated venom peptide bank was 9 months. The high-throughput pipeline and the results depicted below were implemented to cope with this time constraint. Peptides were purified, characterized and prepared for functional tests following a multi-step process in a 96 well plate format. Starting from synthesised genes encoding animal venom peptides, DsbC-His-peptide fusions were purified from crude lysates using an automated nickel affinity procedure. The target peptides were further isolated on C18 resin after cleavage of the DsbC fusion partner by TEV protease. Correct mass, oxidation state and concentration of the resultant peptides were determined by LC–MS. Finally, the concentration of the oxidized venom peptides were adjusted to 10 μM (or 1 μM), aliquoted in multiplates and frozen for the drug discovery process.

### Generation of a library of *E. coli* expressing plasmids encoding 4992 venom peptides

**Fig. 1:**
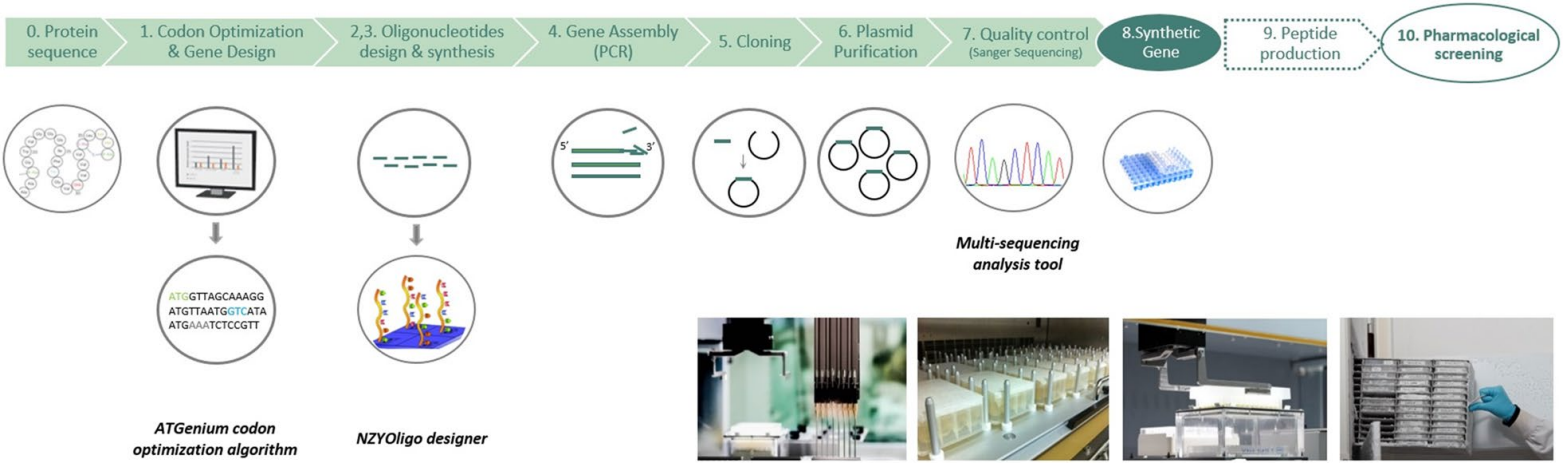
HTP gene synthesis platform used to produce 4992 synthetic genes encoding venom peptides. This pipeline includes 7 steps that allow the successful synthesis of multiples of 96 genes. The *first step* corresponds to gene design and codon optimization; from multiple peptide sequences, DNA sequences are designed and optimized for expression in *E. coli*, using the ATGenium codon optimization algorithm. In *steps 2, 3* and *4* oligonucleotides required for gene assembly are designed using the NZYOligo designer, synthesised and assembled by PCR using optimal conditions, respectively. Synthetic genes are cloned using NZYTech LIC protocol into the *E. coli* expression vector pHTP4. Bacterial transformation and DNA preparations are accomplished using high-throughput protocols. DNA sequences are checked for the presence of sequence errors using a high-throughput sequencing analysis tool. All steps are automated using a Tecan liquid handling system. The plate containing the 96 clones (*step 8*) is ready to go through the peptide production pipeline

**Fig. 2:**
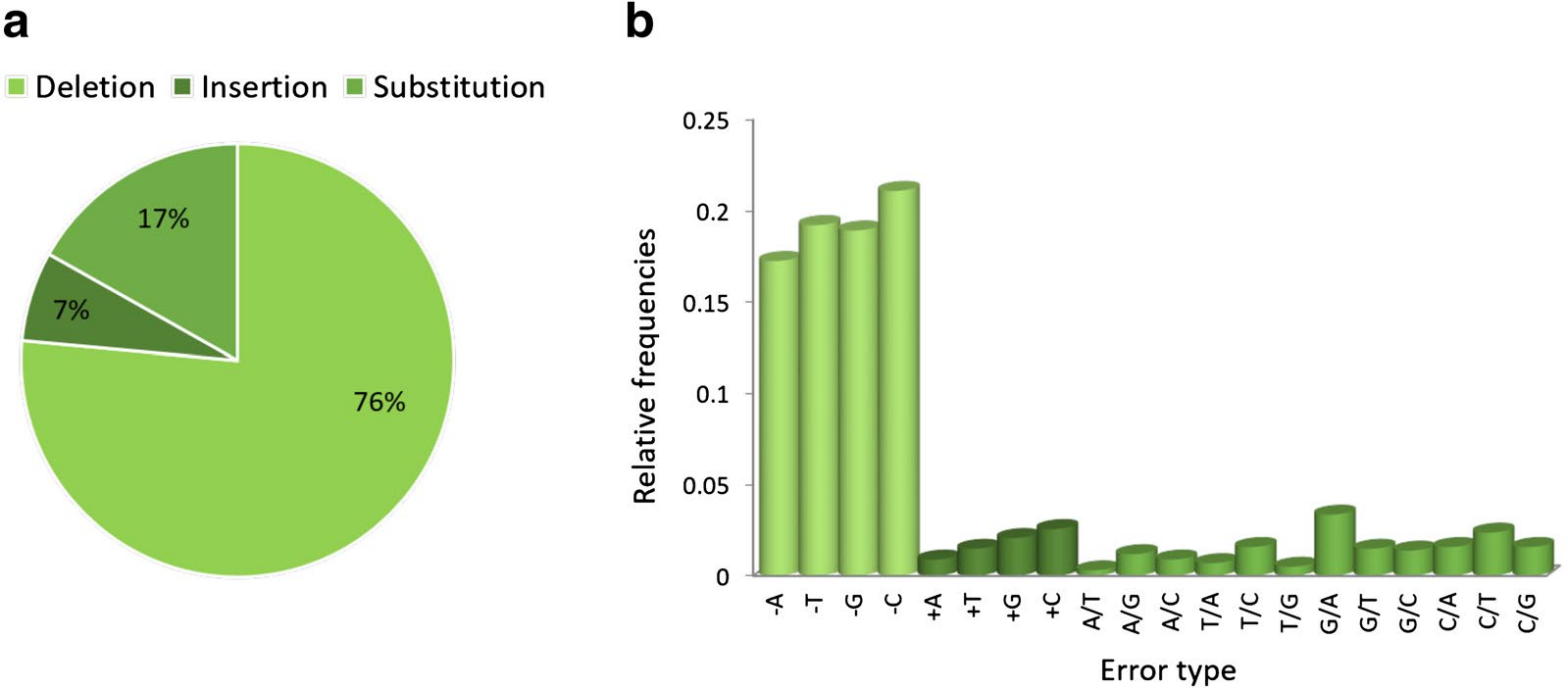
Errors observed during the synthesis of 4992 genes encoding venom peptides. In *Panel* **a** the percentage of types of errors identified in the genes is described. In *Panel* **b** the type of mistakes that were observed are specified

A previously optimized high-throughput platform, described in the accompanying paper [13], was used to synthesise 4992 genes encoding animal venom peptides. The platform is automated for the majority of its steps using 24 or 96 well plates, a standard high-throughput liquid-handling Tecan robot and various bioinformatics tools. A schematic representation of the platform is presented in Fig. 1. The primary sequences of 4992 reticulated peptides originated from 201 venomous animal species were used to design genes for optimal expression in *E. coli*. The algorithm was set to lead to a similar incorporation of the two cysteine codons, as suggested by data presented in the accompanying paper [13]. Genes contained an average GC content of 49% and an average CAI of 0.92 (Table 1). Oligonucleotides used to assemble the gene library were designed to have an overlap region of 20 bp and a gap of also 20 bp, while having a maximum length of 60 bp. On average 6 primers were required to assemble each nucleic acid and genes had an average size of 220 bp (Table 1). Individual genes were PCR assembled and after nucleic acid purification directly subcloned into pHTP4 expression vector using a LIC method. No methodology was implemented to correct eventual mistakes arising during gene synthesis. The robustness of the pipeline was demonstrated when plasmid DNA was sequenced to verify gene integrity. After screening only one colony per cloning reaction, 3818 of the genes (76.5%) were observed to be correct, meaning that for more than 75% of the gene synthesis reactions it was possible to easily recover a gene that accumulated no mutations during nucleic acid assembly. Thus, a second colony was inoculated for 1174 LIC reactions for which no correct clones were obtained in the first screen. When the second colony was screened, 809 of the genes (16.2%) were found to be correct. Finally, for 365 genes (7.3%) it was necessary to pick a third colony to obtain a correct nucleic acid. Taken together the data revealed that it was necessary to screen an average of 1.3 clones to obtain the correct gene. Therefore, this gene synthesis platform exhibited an error rate of 1.06 errors/kb. The majority of the detected errors were deletions (76%), as is expected from the oligonucleotide synthesis methodology [17]. Insertions were less common (7%) while substitutions represent 17% of the identified errors (Fig. 2). The most frequent substitution identified in incorrect genes was a G for an A. Deletions or insertions of a C were the most represented ones while the nucleotide A was less prone to be erroneously inserted or deleted (Fig. 2).

**Table 1:** Properties of the 4992 genes synthesised in this project

	Length (bp)	GC content (%)	Number of primers	Codon Adaptation Index (CAI)
Mean (±SD)	220 ± 54	49 ± 4	6 ± 1.6	0.92 ± 0.04
Maximum	413	58	10	0.94
Minimum	137	42	4	0.8

### Generation of a library of 2736 oxidized venom peptides for drug discovery

**Fig. 3:**
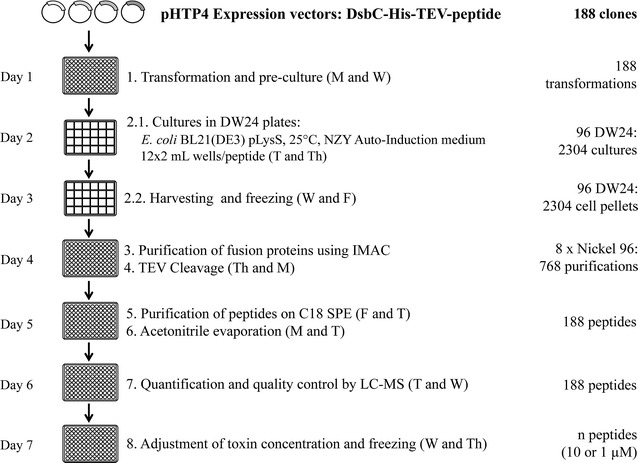
Schematic representation of the high-throughput pipeline used for the production of recombinant venom peptides in *E. coli*. Week schedule; Even numbered plates start on Mondays, odd numbered plates on Wednesdays. Between *bracket* are the days of the week (M, Monday; T, Tuesday; W, Wednesday; Th, Thursday; F, Friday)

In the accompanying paper [13], the most critical parameters required for the production of oxidized disulphide-reticulated peptides in *E. coli* were optimized. Here, this high-throughput protocol was assembled to produce, from a library of 4992 plasmids, a maximum number of oxidized recombinant target peptides in a 9 month period. The platform was mostly automated on a Tecan Freedom Evo liquid-handling robot (Switzerland) using 24 or 96 well plates. The process is made of roughly eight steps. A schematic representation of the pipeline used for the recombinant production of animal venom peptides in *E. coli* is presented in Fig. 3. To cope with the timeline of the project, the purification pipeline (96 peptides) was run twice every week; the team of 3 people performed 2304 cultures, 768 nickel purifications, 188 SPE and 188 LC–MS every single week. At the end of the production (9 months), each peptide had been through the production pipeline only once. The team of three people had successfully implemented a complex process made of eight steps taking from seven to eight days (from the initial culture transformation to the frozen normalized peptide bank; see Fig. 3). This effort represents, altogether, more than 60,000 single cultures, 5000 affinity purifications and 5000 reverse phase purifications followed by 5000 LC–MS quality controls and quantification. This is, to our knowledge, an unprecedented production effort in such a short time frame.

From the 4992 peptides that entered the pipeline, 4963 (99.4%) were analysed by LC–MS. The 29/4992 peptides (0.6%), lost before the end of the production, failed to give viable colonies or cultures. Due to time constraints these cultures were not reproduced. The cultivable 4963 peptides (99.4%) were successfully purified by IMAC. Among these, 66 peptides (1.3%) were initially lost during the purification (clogged wells or cross contamination due to partial flooding of a neighbouring well). These 66 peptides were combined in a salvage experiment. Thus, the cultures were reproduced and the 66 peptides reached the LC–MS step on a second batch. Together, from the 4992 peptides that entered the pipeline, 4963 went up to the affinity purification step and 2736 (55%) were produced with the correct peptide molecular weights, corresponding to their fully oxidized isoforms (Additional file 3: Table S3; Additional file 4: Figure S1). The peptide molecular weights were controlled twice during the process, at the end of the production by the in-house LC–MS system and after the aliquoting and freezing of the bank by ESI or MALDI MS (University of Liège). The second aliquot (after one freeze–thaw cycle) confirmed in most cases that during the re-organization of peptides in micro-titer plates no or very few position errors occurred. The validation of the bank by Liège was made in 6 batches of 6 plates, the result of the first batch is displayed in Additional file 4: Figure S1. This batch is representative of the average results on the full bank. The mass of the oxidized peptide was correct in 98% of the cases (Additional file 4: Figure S1).

Confirming that the peptides are oxidized is quiet easy by mass spectrometry. However, the determination of their correct connectivities is far from being trivial. The classical strategies for resolving the disulfide connectivities in proteins rest on a combination of liquid chromatography, mass spectrometry and NMR. In the case of the accompanying article and the previous study [11], working with known and well characterized toxins, everytime a toxin was purified, the mass spectrometry (coupled with Liquid Chromatography) not only confirmed the exact oxidized mass of all species, but also the correct connections of the disulfide bridges after cleavage by proteases. In these three studies ([11], this article and the accompanying article), at the end of the purification, if a toxin peak was detected and quantified, it was always corresponding to an oxidized form of the toxin (and when this toxin was known, to its native form). The reduced forms of the peptides were never detected, probably due to the precipitation of incorrectly oxidized peptides during cleavage and acidification steps. Either the toxin survived the harsh pipeline or it was lost for good.

In the case of the VENOMICS database, in fact the natural folds and exact connections of the disulfide bridges are unknown so nobody can guaranty that the toxins that got purified for this peptide bank have the natural fold and connection. On the other hand, as it was stated earlier, if it was not the case these peptides probably wouldn’t have survived the pipeline and freezing cycle. The VENOMICS database contains 25,000 new toxins for which a subset of 5000 were produced by recombinant expression. These 5000 sequences were selected after merging the dataset of the transcriptomics analysis (Sistemas Genomicos, Valencia, Spain) and proteomics (University of Liège) to have a better confidence that they represent real full length native venom peptides. Transcriptomics technology does not give information on the connection of the disulfide bridges and for the proteomics analysis, even if the team developed, using mass spectrometry, a protocol to sequence full de novo venom peptides in an automatic manner and applied this to 180 venoms, the workload made impossible the case to case fragmentation of each toxins that would have been necessary to try to identify most of the connections. Even if this would have been possible; the confirmation of these data would have requested big quantities of venoms and natural toxins to reproduce and compare the results.

The characteristics of each peptide and the output of their production are presented in Additional file 3: Table S3. From the 2736 peptides of the bank, 2174 (~80%) had a concentration above 5 µM and were aliquoted in the “10 µM bank” (28 plates), while the remaining 562 (~20%), with a concentration ranging from 1 to 5 µM, were aliquoted in the “1 µM bank” (8 plates). Among the 10 µM bank, 1363 (~63%) were purified at a concentration above 20 µM. The resulting bank contains to date, the biggest and most diverse collection of recombinant animal venom peptides available for drug discovery screening. These statistics demonstrate the extreme robustness of the production and purification pipeline.

Out of the 4963 purified fusion peptides, 4843 (97.6%) DsbC-His-toxins could be purified in milligram quantities (per litre of culture) after IMAC purification. In theory, with a 100% cleavage efficiency and recovery, these should have allowed the purification of peptides in the levels required for the drug discovery pipeline. For the 2736 peptides of the bank, the average yield of purified fusion protein per litre of culture was 186 mg (with a maximum yield of around 500 mg/L). These data are in accordance with the result obtained in the accompanying paper [13]. Thus, the average yield per litre reflects the purification of a total of 4.4 mg of fusion peptide using the pipeline (from 24 mL culture). However, to avoid compromising the throughput of the pipeline, yields of purified fusion peptides were not determined on Caliper or LC–MS, which could have provided information about presence of truncated derivatives of the proteins. Concentration of the purified protein was quantified only by OD at 280 nm. These concentrations are, therefore, probably overestimated for a portion of the population of the DsbC-His-peptides. Indeed, in the accompanying paper [13] we identified, by Caliper analysis of the purified fusions, that in some cases the correct population (DsbC-His-peptide) was contaminated by truncated forms of the fusion protein (DsbC-His alone). Notwithstanding these observations, from the 2736 peptides aliquoted for drug discovery (average mass of 5935 Da representing 17.6% of the full fusion), the average concentration of purified recombinant peptide obtained at the end of the last purification step was 65.7 µM (in 250 µL), leading to an average yield of 3.91 mg of peptide per litre of culture (97 µg/16.4 nmoles in the 250 µL, 0.39 mg/mL concentration, from the 24 mL culture scale). Therefore, the overall average recovery of peptides (11.9%), turned out to be below the values found in the accompanying paper [13] where the average recovery from the pHTP4 vector was 17%. This could be explained by several reasons. First, as explained above, the yield of purified fusion is in some cases over-estimated. Secondly, for some peptide families that were not present in the previous tests (see accompanying paper), the TEV cleavage might not be complete due to non-optimal cleavage conditions (TEV ratio, DTT concentration in the buffer, etc.). In addition, following the present protocol, peptides are purified from the DsbC-His fusion by a C18 purification step that was not present in the pipeline described previously [13]. Finally, peptides with non-native inter-chain bonds do precipitate in this rather harsh protocol (all steps done at room temperature, quick acidification, etc.). This is confirmed by the fact that the non-correctly oxidized or mixed forms (intermolecular disulphide bridges) peptides were never detected on the LC–MS.

### The pipeline is efficient for the production of venom peptides independent of animal origin, peptide length, cysteine pattern or number of disulphide bridges

**Fig. 4:**
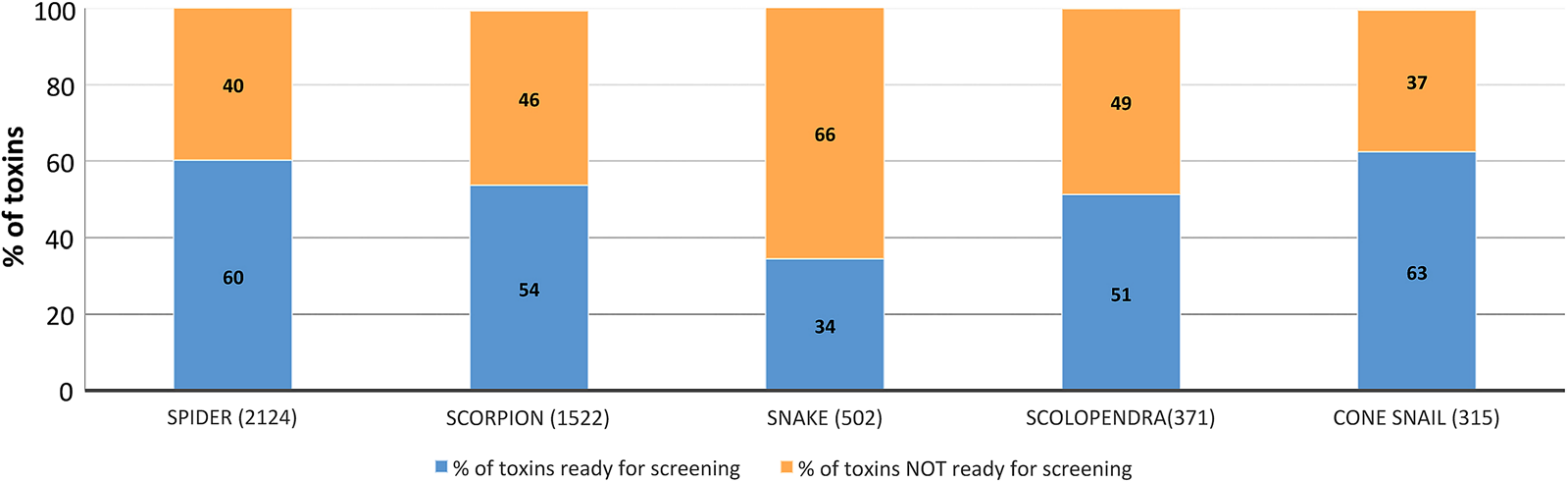
Effect of venom peptide origin in the success rate of production. The number of peptides analysed for the different animals is presented in brackets. In *orange* percentage of peptides not produced successfully. In *blue* percentage of peptides produced in sufficient quantities for screening

**Fig. 5:**
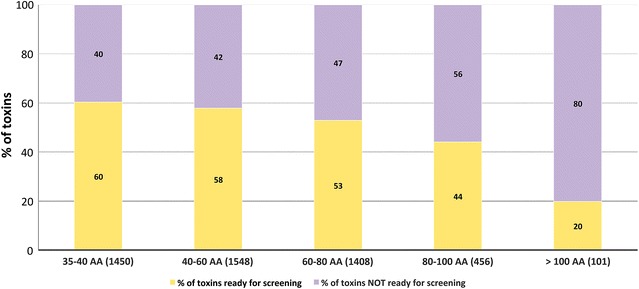
Effect of peptide length in the success rate of production. The number of peptides analysed for the different classes of peptide length is presented in *brackets*. In *purple* percentage of peptides not produced successfully. In *yellow* percentage of peptides produced in sufficient quantities for screening

One of the objectives of this study was to set up a collection of peptides representing the natural diversity in animal venom. The panel selected here was issued from a wide variety of animals with variable peptide lengths (35–120 amino acids), number of disulphide bridges (1–9) and cysteine patterns (84 different cysteine patterns). The 4992 peptides that were selected for the recombinant production were originally identified from 201 animal species. The most represented species were spiders (2124 samples, 43%), scorpions (31%), snakes (10%), centipedes (scolopendra) (7%) and cone snails (6%). Several other species were also present (ants, anemone and various insects, 3% altogether, with an average success rate of 60%). While peptides sized 35 amino acids or longer were produced recombinantly, within VENOMICS smaller peptides were produced via chemical synthesis. Since they contain mostly small peptides, the synthesis focussed on cone snail (56%), spider (29%) and scorpion (8%) peptides. The success rate of the production of recombinant animal venom peptides is displayed in Fig. 4. Overall the data suggest that with the exception of peptides from snakes, with a success rate of only 34%, the source origin had little impact on the capacity to obtain peptides recombinantly. Data presented in Fig. 5 suggest that although the pipeline successfully expressed the majority of peptides, the success rate decreased with increasing peptide length. The data confirm a drastic decrease in the capacity of producing recombinant peptides when sizes are bigger than 100 residues. However, these large size peptides were underrepresented in the 4992 bank as they correspond to 2% of the plasmid bank. The snake peptides selected for the bank are on average among the longer peptides of this study which probably explains why the pipeline was not as adequate for these venom peptides than the ones from other organism.

**Fig. 6:**
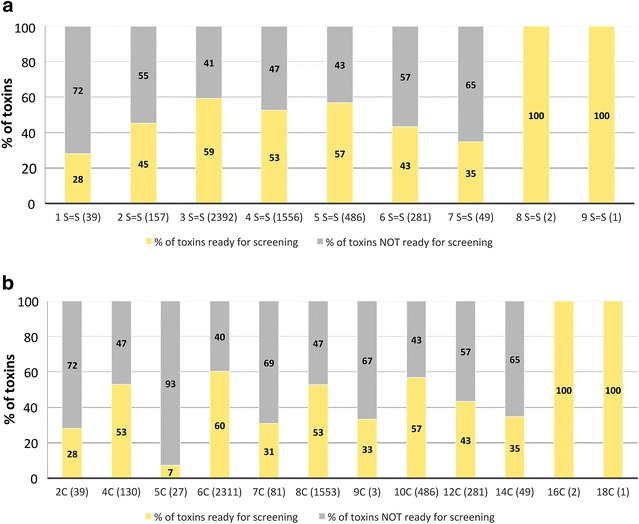
Effect of number of disulfide bridges (*Panel* **a**) and number of cysteine residues (*Panel* **b**) in the success rate of production. The number of peptides analysed in the two different cases is presented in *brackets*. In *grey* percentage of peptides not produced successfully. In *yellow* percentage of peptides produced in sufficient quantities for screening

Within polypeptides, cysteine patterns reflect the number and distribution of cysteines along the primary sequence. From the 84 cysteine patterns analysed in this work, the majority were successfully produced as correctly folded toxins (67/84, see Additional file 5: Figure S2). Except for 18 patterns (representing 0.6% of the targets) where no oxidized peptide could be detected, the other patterns exhibited some success demonstrating the rather wide spectrum capacity offered by the DsbC-fusion expression system. These patterns span from the shortest “C–C” peptides (one disulphide bridge, 39 peptides; 28% successful production), to peptides with 8 and 9 disulphide bridges (that could be fully recovered). Some patterns that are highly represented (see Additional file 5: Figure S2, in boxes) could be tentatively associated with putative disulfide bridge reticulation, and therefore with peptide 3D structures. For example, C–C–CC–C–C (1221 occurrences, 67% success rate) is the most abundant cysteine pattern found in this study and it can easily be associated with the inhibitor cystine knot (ICK) motif, highly present in spider and conus venom peptides. While only one has been described in the past for scorpions, this study includes 15 non-redundant new scorpion sequences. Three finger snake peptides can be recognized with the following patterns: C–C–C–C–C-CC–C (471 occurrences 32% success) and C–C–C–C–C–C–C–CC–C (23 occurrences, 48% success), for the ones having an extra bond on one of the three fingers. Three-finger-fold toxins are one of the most successfully produced toxin super-families from snake venom. The patterns C–C–C–C–C–C (937 occurrences; 52% success) and C–C–C–C–C–C–C–C (424 occurrences, 51% success) indicate a Csα/β fold known in scorpion toxins. Finally, C–C–C–C (122 occurrences, 53% success) could well stabilize the secondary structure found in anemone peptides (Csα/α) and was rarely described for scorpion venom peptides to date. The pipeline was more efficient at producing peptides with 3, 4 or 5 disulphide bridges, which represent 70% of the peptide bank (Fig. 6a). The success rate slightly decreases for more complex peptides (6 and 7 bridges). Remarkably, the three most complex peptides, containing 8 (2 peptides) and 9 (1 peptide) disulphide bridges, were successfully produced while the pipeline turned out to be comparatively inefficient for the shorter peptides. Only 28% of peptides containing only one disulphide bridge were obtained recombinantly. A detailed analysis of the 4992 primary sequences comprising the peptide bank revealed the presence of 99 peptides with an odd number of cysteines, a feature not common in the venom peptide field [9, 18]. Data, presented in Fig. 6b, revealed that for peptides containing an odd number of cysteines, the success rate of production was much lower when compared with peptides containing a paired number of cysteines. This observation is intriguing and more work is required to determine under which quaternary structure, monomeric, dimeric or multimeric, these peptides exist in the venom.

**Fig. 7:**
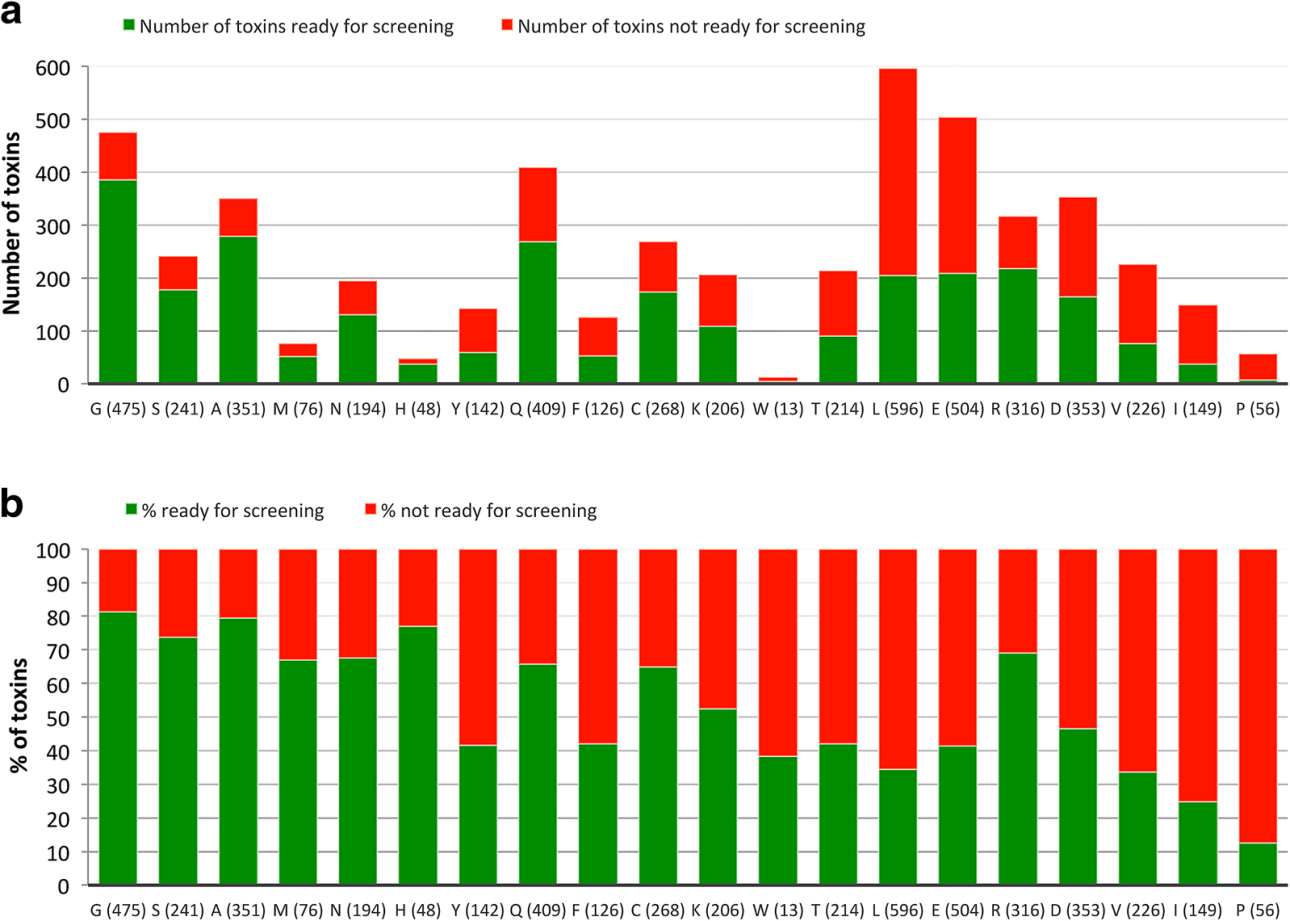
The nature of the N-terminal residue in native venom peptides affects the success rate of production. *Panel* **a** displays the number of peptides containing the 20 different amino acids at the N-terminus. In *Panel* **b**, the percentage of peptides produced or not produced is displayed. The number of peptides analysed is presented in *brackets*. In *red* percentage of peptides not produced successfully. In *green* percentage of peptides produced in sufficient quantities for screening

The N-terminal residues of venom peptides can contribute to the ligand binding site. It is, therefore, possible that introduction of a single, extra residue, at the N-terminus of recombinant peptides may affect their biological activity [19]. Data presented in the accompanying paper [13] suggested that, with exception of proline, all residues could be accommodated in the P1 position of the TEV protease recognition site while maintaining some efficacy of processing. Thus, in this study, no extra residue was engineered at the N-terminus of the recombinant peptides to improve TEV cleavage efficacy. Analysis of the primary sequences of the 4992 peptides revealed that all 20 amino acids can be found at the N-terminus of the native peptide sequence (Fig. 7). The most represented N-terminal amino acids in venom peptides of the 4992 sequences are leucine, glutamate and glycine (around 10% of the bank for each one) (Fig. 7a). N-terminal cysteines, which should also be involved in disulphide-bonding, are relatively abundant in the population while peptides starting with a proline, histidine or tryptophan are very rare (1% or less). The relationship between the nature of the N-terminal residue and the capacity to produce the corresponding peptide was analysed. The data, presented in Fig. 7b, suggested a similar trend of cleavage efficiency when compared with data presented in the accompanying paper [13]. Thus, this observation indicates that the folding of the peptide had little impact on the cleavage efficiency, which is more affected by the nature of the N-terminal residue of the peptide. Strikingly, cleavage was very effective for peptides containing a cysteine in position 1, suggesting that presence of residues participating in disulphide bridges at the N-terminus of the polypeptide does not interfere with the TEV protease access. In addition, even if uncommon (1.1%), it was possible to cleave off some peptides containing a proline at position 1 (13%). Thus, it is possible that in particular proteins the primary sequence downstream the proline residue allows it to assume a conformation that is more permissible for proteolytic attack.

## Discussion

There is an urgent need to develop a novel model for modern drug discovery. Monoclonal antibodies have recently become increasingly attractive therapeutics, delivering important results in the treatment of several major disorders including autoimmune disease, cancer, inflammation, cardiovascular and infectious diseases [4, 20]. The fact that antibodies have become captivating therapeutic agents motivated a recent interest in other biological molecules, and particularly peptides, as valid leads for innovative drugs [21]. Animal venoms constitute a vast and essentially untapped resource of novel biologically active molecules and thus should play a prime role in modern drug discovery [22–24]. Venoms evolved in a wide variety of invertebrates (e.g., sea anemones, corals, jellyfish, marine molluscs, spiders, scorpions, hymenopteran insects and marine worms), as well as vertebrates, such as snakes [25]. It is now well established that venom peptide targets are involved in various human pathologies such as pain, cancer, neurodegenerative diseases, cardio-vascular diseases, diabetes, obesity and depression. Thus, animal venoms constitute vast libraries of pharmacologically active disulphide-reticulated peptides, which have evolved to be highly selective receptor-targeted molecules presenting low immunogenicity and high stability [4]. However, replicating Nature’s venomic diversity in vitro, to generate large collections of bioactive reticulated peptides that could be pharmacologically screened to identify novel drug leads is still, mostly, an unrealized prospect. Genuinely, the recombinant expression and refolding of reticulated peptides has been a major limiting factor in the use of venoms for drug discovery.

With the aim of exploring the huge biodiversity presented by animal venoms, we have developed and optimized a high-throughput pipeline for the production of large libraries of bioactive recombinant venom peptides. Data presented here reveals that *E. coli* is an effective heterologous host to express large numbers of recombinant fully-oxidized venom peptides. The genes encoding approximately 5000 venom peptides from different sources were de novo synthesised with a codon usage optimized for expression in *E. coli*. A total of 1100 kb of DNA was synthesised during this project. The gene synthesis method employed here exhibited a low error rate of 1.06 errors/kb, which is similar or slightly better when compared with other gene synthesis methodologies based on PCR or ligation assembly [26–29]. Approximately 25% of the genes were found incorrect after screening only one clone per gene. However, these genes were easily recovered by screening a second or eventually third clone, revealing the capacity of this high-throughput pipeline to synthesize genes in a large scale. As observed here, regardless of the synthesis method, incorrect bases are likely to be incorporated into the gene sequence during assembly. These arise mostly due to errors accumulated during the chemical synthesis of the oligonucleotides [17]. Thus, the most common errors observed during de novo gene synthesis are deletions that result from the incorporation of truncated versions of the oligonucleotides used for gene assembly. Typically, these errors require removal to retrieve the integral DNA sequence [27, 30], although this process introduces complexity in the gene synthesis process, which most of the time is not compatible with high-throughput methods. Various strategies were developed to reduce encoded errors, including use of enzymatic mismatch cleavage [26, 31], mismatch-binding proteins [32] and site-directed mutagenesis [30]. Since genes encoding venom peptides are relatively small (average size of 220 bp) a low error rate was expected and, therefore, an error correction technology was not employed in this pipeline.

Here, a high-throughput platform was used to establish a large library of recombinant venom peptides for drug discovery. The library contains 2736 different correctly oxidized peptides of different origins, validating the capacity of *E. coli* to produce bio-active disulphide-reticulated peptides. Due to the workload of the project, each peptide has been through the pipeline only once, it has to be noted that therefore no individual optimization were performed to increase the yield or to try to salvage failures based on the analysis of cysteine pattern, source organism, etc. For a project with less peptides one could reasonably expect to increase yields or to find protocols that would be more specific to some families of peptides. The accompanying paper could help to decide which alternative protocol should be tried first. It clearly demonstrates that the choice of codon usage, of the fusion and compartment of expression, of cleavage buffer to cite few, turned out to have a major effect on the results. For the peptide bank produced here, the major factor affecting the capacity of bacterial cells to express animal venom peptides is the peptide length, as production efficacy dramatically decreases for peptides with more than 100 amino acids. Overall the percentage of correctly oxidized peptide is much reduced when compared with the production of the DsbC-His-fusion peptide derivative, suggesting that a considerable fraction of the peptides are not properly folded when produced in the fusion form. Nevertheless, using the DsbC fusion partner and directing the expression to the periplasm allows significant levels of biologically active venom peptides to be obtained in *E. coli*. In addition, it is possible that a significant number of peptides were not properly produced due to difficulties in cleaving the DsbC fusion tag. This may be true for peptides containing a proline, isoleucine or valine at the N-terminus, as data presented in the accompanying paper [13] revealed that TEV protease was not completely efficient in cleaving sequences containing those residues at position P1′. For this subset of peptides inclusion of an extra glycine residue at the N-terminus might lead to a higher recovery of cleaved peptide. However, it is also possible that presence of this extra residue might affect the biological activity of the resulting recombinant animal venom peptides.

## Conclusions

The natural venom collection may be comprised of up to 40,000,000 different peptides and can be seen as an attractive source of stable and evolutionarily fine-tuned highly selective molecules for drug discovery. In the scope of the VENOMICS project a sequence database of ~25,000 novel genes encoding venom peptides was produced based on transcriptomic and proteomic data collected in 201 different animal species. A subset of ~5000 peptides with more than 35 amino acids and representative of the natural diversity observed in the database was selected for recombinant production in bacteria following protocols established in the accompanying paper. Here we describe the high-throughput production of a unique library of recombinant venom peptides expressed in *E. coli*. The library contains 2736 animal venom peptides that were shown to be oxidized. Within VENOMICS, the library described here was screened on several therapeutic targets including ionic channels and G-Protein Coupled Receptors. The data lead to the identification of dozens of different hits in the majority of the molecular targets tested. Unfortunately, the toxin sequences and the target names under studies by the VENOMICS consortium cannot be disclosed in publications. Because the peptides produced here were novel, the ‘correct’ disulphide-bonding patterns and bioactivities are not explicitly known, but given the fact that only oxidized toxins were detected during the quality control of the process and the already significant success rate of the screening, it is inferred that the correctly-oxidized active conformations were reached. Overall data presented in this paper confirms that *E. coli* is an effective host for the production of large libraries of venom peptides for drug discovery programs. While the exact sequence of the 4992 peptides of this study remains confidential, the high-throughput protein production process described here could be adapted to the generation of innovative libraries of different peptides and protein families with interest for drug discovery.

## Additional files

**Additional file 1: Table S1.** Animal groups and number of species within each animal group used for the selection of the 4992 peptides produced recombinantly within this study.

**Additional file 2: Table S2.** Codon frequency used to design 4992 optimized encoding venom peptides.

**Additional file 3: Table S3.** Additional table.

**Additional file 4: Figure S1.** Quality control of the produced toxins. (A) 50 mM ammonium formiate was added to each 96-well plate containing the peptides of the bank (80 toxins/plate). The plate was inserted into a automatic injector (Waters iClass), adjusted to inject 1µL of toxin every 10 min, into a TOF mass spectrometer (MicroTOF, Bruker) through an electrospray source. The signals were deconvoluted giving access to the molecular masses of the produced toxins. The 36 plates were analyzed in 6 × 6 plate batches. Experimental masses were finally compared to the theoretical ones. B Rate of success obtained for this batch, 97.9% of this batch massses are correct (oxidized theoretical mass) with an error ± 0.4 Da. These values are representative of the full library (MS validation Batch 1, n = 480 toxines). The analyzed toxins of Batch 1 are spread over a large mass range, from 4 to 10 kDa.

**Additional file 5: Figure S2.** Influence of cysteine pattern in the success rate of production. The 4992 venom peptides produced in this project represent 84 different cysteine patterns. The figure correlates the type of cysteine pattern with percentage of peptides efficiently produced in *E. coli* (yellow) or not efficiently produced in the bacterial host (grey).

## Abbreviations

CAI: codon adaptation index
DTT: dithiothreitol
DsbC: disulfide oxidoreductase C
FP7: framework program 7 (European funding)
HTP: high-throughput
LIC: ligation independent cloning
TEV: tobacco etch virus
