# Supplementary material for: High-throughput expression of animal venom toxins in Escherichia coli to generate a large library of oxidized disulphide-reticulated peptides for drug discovery
Source: Microb Cell Fact. 2017 Jan 17;16:6. doi: 10.1186/s12934-016-0617-1 (PMC5242012; doi:10.1186/s12934-016-0617-1)
Supplement: Supplementary file 1 — Additional file 1: Table S1. Animal groups and number of species within each animal group used for the selection of the 4992 peptides produced recombinantly within this study. [file 12934_2016_617_MOESM1_ESM.docx]

**Table S1-** Animal groups and number of species within each animal group used for the selection of the 4992 peptides produced recombinantly within this study.

| Venomous animal | Families | Number of species |
| --- | --- | --- |
| Snakes | *Elapidae*, *Viperidae*, *Atractaspididae* | 38 |
| Scorpions | *Buthidae*, *Euscorpiidae*, *Hemiscorpiidae*, *Scorpionidae*, *Scorpionidae*, *Vaejovidae* | 41 |
| Cone snails | *Conidae* | 40 |
| Spiders | *Agelenidae, Araneidae,Ctenidae , Dipluridae, Lycosidae, Nephilidae,Sparassidae, Theraphosidae, Theridiidae* | 46 |
| Fishes | *Potamotrygonidae*, *Plotosidae*, *Siluridae*, *Synanceiidae* | 9 |
| Hymenoptera | *Apidae*, *Vespidae* | 8 |
| Scolopendra | *Scolopendridae* | 5 |
| Terebra/mitre | *Mitridae*, *Terebridae* | 4 |
| Cnidarians | *Actiniidae*, *Cassiopeidae, Stichodactylidae* | 3 |
| Octopus | *Octopodidae* | 3 |
| Ants | *Formicidae*, *Myrmicinae* | 2 |
| Rays | *Potamotrygonidae* | 1 |
| Lizards | *Helodermatidae* | 1 |
| Total |  | 201 |
