# Supplementary figures and images for: High-throughput expression of animal venom toxins in Escherichia coli to generate a large library of oxidized disulphide-reticulated peptides for drug discovery
Source: Microb Cell Fact. 2017 Jan 17;16:6. doi: 10.1186/s12934-016-0617-1 (PMC5242012; doi:10.1186/s12934-016-0617-1)

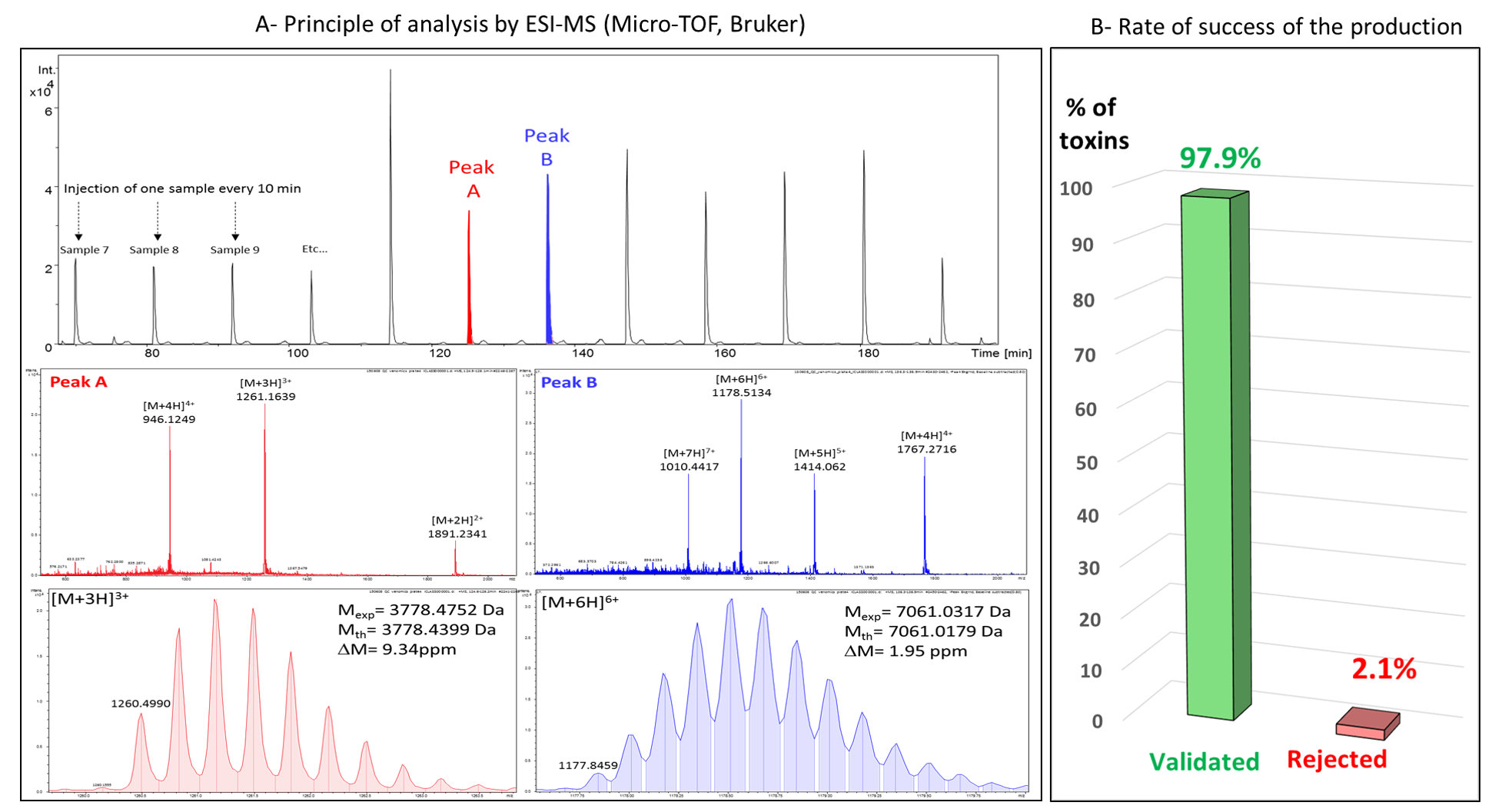

Supplement: Supplementary file 4 — Additional file 4: Figure S1. Quality control of the produced toxins. (A) 50 mM ammonium formiate was added to each 96-well plate containing the peptides of the bank (80 toxins/plate). The plate was inserted into a automatic injector (Waters iClass), adjusted to inject 1µL of toxin every 10 min, into a TOF mass spectrometer (MicroTOF, Bruker) through an electrospray source. The signals were deconvoluted giving access to the molecular masses of the produced toxins. The 36 plates were analyzed in 6 × 6 plate batches. Experimental masses were finally compared to the theoretical ones. B Rate of success obtained for this batch, 97.9% of this batch massses are correct (oxidized theoretical mass) with an error ± 0.4 Da. These values are representative of the full library (MS validation Batch 1, n = 480 toxines). The analyzed toxins of Batch 1 are spread over a large mass range, from 4 to 10 kDa. [file 12934_2016_617_MOESM4_ESM.png]

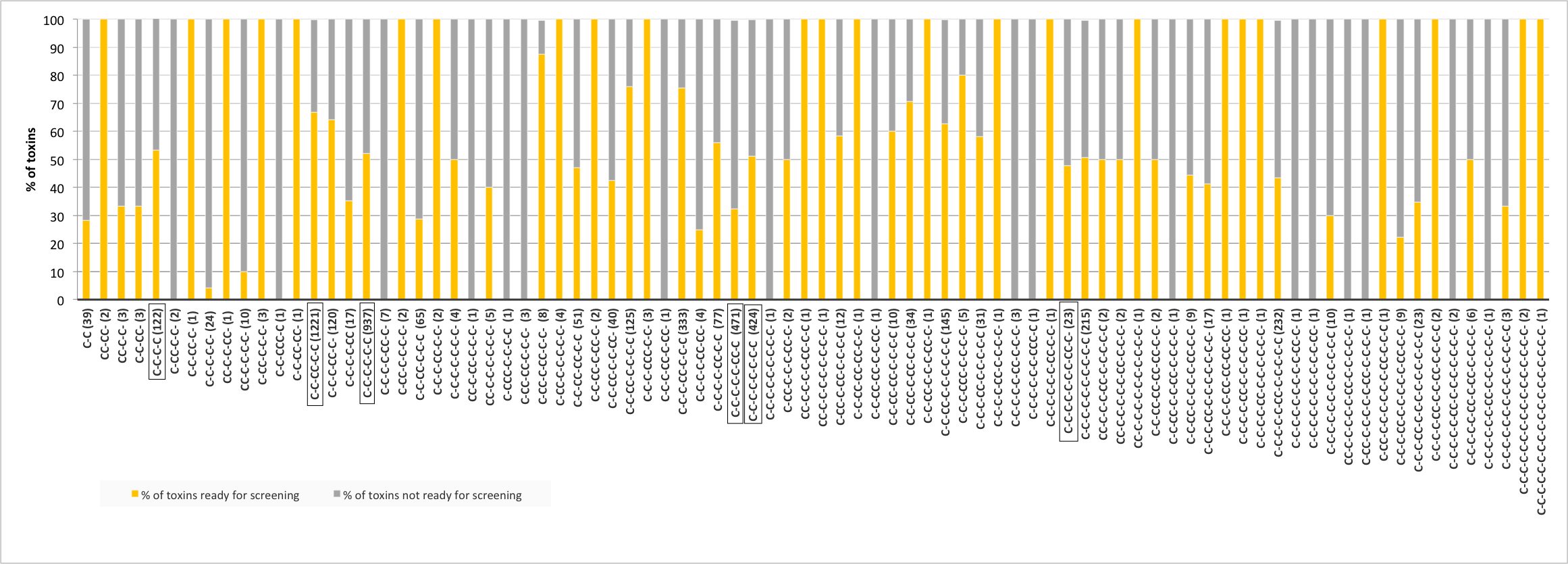

Supplement: Supplementary file 5 — Additional file 5: Figure S2. Influence of cysteine pattern in the success rate of production. The 4992 venom peptides produced in this project represent 84 different cysteine patterns. The figure correlates the type of cysteine pattern with percentage of peptides efficiently produced in E. coli (yellow) or not efficiently produced in the bacterial host (grey). [file 12934_2016_617_MOESM5_ESM.png]
